# Kea, *Nestor notabilis*, achieve cooperation in dyads, triads, and tetrads when dominants show restraint

**DOI:** 10.3758/s13420-021-00462-9

**Published:** 2021-02-02

**Authors:** R. Schwing, E. Meaux, A. Piseddu, L. Huber, R. Noë

**Affiliations:** 1 Comparative Cognition Unit, Messerli Research Institute, University of Veterinary Medicine Vienna, University of Vienna, Medical University Vienna, Veterinärplatz 1, 1210 Vienna, Austria; 2 Haidlhof Research Station, University of Veterinary Medicine, University of Vienna, Haidlhof 204, 2540 Bad Vöslau, Austria; 3 Psychologie, Université de Strasbourg, 12 Rue Goethe, 67000 Strasbourg, France; 4 College of Forestry, Guangxi University, No. 100 Daxue Road, Nanning, Guangxi 530005 People’s Republic of China

**Keywords:** Tetradic cooperation, Coordination, Kea, Parrot, Restraint, Tolerance, Dominance, Rank distance, Affiliation

## Abstract

Animal cooperation in the wild often involves multiple individuals that must tolerate each other in close proximity. However, most cooperation experiments in the lab are done with two animals, that are often also physically separated. Such experiments are useful for answering some pertinent questions, for example about the understanding of the role of the partner and strategies of partner control, but say little about factors determining successful cooperation with multiple partners in group settings. We explored the influence of dominance, rank distance, tolerance, affiliation, and coordination by testing kea parrots with a box requiring two, three, or four chains to be pulled simultaneously to access food rewards. The reward could be divided unevenly, but not monopolized completely. Eventually dyadic, triadic, and tetradic cooperation tasks were solved, showing that non-human animals are capable of tetradic cooperation in an experimental setup. Starting with two chains, we found that in a dyad monopolization of the box by the highest-ranking bird was the largest obstacle preventing successful cooperation. High-ranking birds learned to restrain themselves from monopolizing the box during a single session in which monopolization was hindered by the presence of a large number of birds. Thereafter, restraint by dominants remained the strongest factor determining success in the first trial in dyadic, triadic, and tetradic setups. The probability of success increased with the degree of restraint shown by all dominant subjects present. Previous experience with the task contributed to success in subsequent sessions, while increasing rank distance reduced success notably in the four-chain setup.

**Supplementary Information:**

The online version contains supplementary material available at 10.3758/s13420-021-00462-9.

## Introduction

Over the last two decades, experiments testing cooperation in animals have been conducted successfully with many different species, including corvids (*Corvus corax –* Asakawa-Haas, Schiestl, Bugnyar, & Massen, 2016; Massen, Ritter, & Bugnyar, 2015; *Corvus frugilegus –* Seed, Clayton, & Emery, 2008; *Corvus moneduloides –* Jelbert, Singh, Gray, Taylor, & Marshall, 2015), parrots (Psittacus Erithacus – Péron, Rat-Fischer, Lalot, Nagle, & Bovet, 2011; Nestor notabilis – Schwing, Jocteur, Wein, Massen, & Noë, 2016; Schwing, Reuillon, Conrad, Noë, & Huber, 2020), primates (*Pongo pygmeus –* Chalmeau, Lardeux, Brandibas, & Gallo, 1997; Cebus apella – Mendres & de Waal, 2000; Visalberghi, Quarantotti, & Tranchida, 2000; *Saguines oedipus –* Cronin, Kurian, & Snowdon, 2005; *Callithrix jacchus –* Werdenich & Huber, 2002; P*an troglodytes –* Hare, Melis, Woods, Hastings, & Wrangham, 2007; Melis, Hare, & Tomasello, 2006), canines (*Canis lupus –* Marshall-Pescini, Schwarz, Kostelnik, Virányi, & Range, 2017; *Canis familiaris*), as well as other mammals (*Crocuta crocuta –* Drea & Carter, 2009; *Elephas maximus –* Plotnik, Lair, Suphachoksahakun, & De Waal, 2011; *Tursiops truncatus –* Jaakkola, Guarino, Donegan, & King, 2018). A large proportion of this work has been conducted with the loose-string paradigm, first implemented with chimpanzees (Hirata, 2003; Melis et al., 2006), but since then utilized with several other species (Asakawa-Haas, Schiestl, Bugnyar, & Massen, 2016; Güntürkün & Bugnyar, 2016; Péron, Rat-Fischer, Lalot, Nagle, & Bovet, 2011; Plotnik, Lair, Suphachoksahakun, & De Waal, 2011; Schmelz, Duguid, Bohn, & Völter, 2017). In this setup two subjects are required to pull on both ends of a string to gain access to an out-of-reach platform with rewards; pulling by only one subject results in the task becoming unsolvable. After obtaining proficiency in the task, the initial delay of one partner’s access to the string for short periods of time forces the other partner to wait to be able to solve the task. Waiting for the partner is usually interpreted as a sign of understanding the need for that partner. While these studies have added invaluable information regarding several species’ ability to show such complex cooperation behavior under laboratory conditions, different authors (e.g., Boesch & Boesch, 1989; Noë, 2006) indicate that natural occurrences of cooperation would not always require a high level of understanding regarding the actions of the partner. Boesch and Boesch (1989) in describing behavior in chimpanzees, suggested four levels of growing complexity with regard to hunting, all of which were considered cooperative as they were all directed at the same prey item: (1) similarity *–* similar actions but without relation in time and space; (2) synchrony *–* similar actions with relation in time; (3) coordination *–* similar actions with relation in time and space; and (4) collaboration *–* different actions that are complementary in nature in working to achieve success. Based on these levels, a subject that waits for its partner in the loose-string paradigm has shown the ability for coordination, or at least synchrony, as the spatial aspect is often artificially restricted by the laboratory setting (although see, e.g., Marshall-Pescini, Schwarz, Kostelnik, Virányi, & Range, 2017, or Schwing et al., 2020, for setups with a spatial aspect by presenting subject(s) with two apparatuses simultaneously). However, cooperation in the similarity category can still lead to a mutual benefit, without the adjustment of behaviors based on the partner’s presence. Noë (2006) defined cooperation as “all interactions or series of interactions that, as a rule (or ‘on average’), result in net gain for all participants” (p. 4) and described “instrumental cooperation” as only requiring an understanding of the association between one’s own actions in a cooperative setting and the eventual benefit gained. However, he also stated that it is unlikely that such a learning mechanism alone could lead to cooperative relationships in a natural setting, and suggested that species that exhibit cooperative behavior would have likely undergone selection for social traits that are instrumental for cooperation to occur. Tolerance is put forth as a trait that in itself can be considered a cooperative investment, by allowing individuals to co-occur in the same space and time, thus allowing for positive associations between action and beneficial outcome to be learned (Petit, Desportes, & Thierry, 1992). A lack of tolerance can lead to dominant individuals acting aggressively towards conspecifics, displacing them and thus preventing cooperation from occurring. Tolerance, or the lack of behavior typical for dominant animals, is therefore instrumental in understanding how achieving cooperation can be learned. Tolerance in a lab setting is generally used to describe the occurrence of co-feeding in artificial and natural shareable food patches *–* notably in the primate literature (Hare, Melis, Woods, Hastings, & Wrangham, 2007; Kasper, Voelkl, & Huber, 2008; Melis et al., 2006; Mendres & de Waal, Mendres & de Waal, 2000; Petit et al., 1992; Suchak, Eppley, Campbell, & de Waal, 2014), but also in cooperation studies with corvids (Massen et al., 2015; Seed, Clayton, & Emery, 2008).

In general, studies of cooperation in a variety of species have shown that dominant behavior among the subjects can prevent successful cooperation, notably when food sources are involved (Hare et al., 2007; Massen et al., 2015; Malini Suchak et al., 2014; Seed et al., 2008; Werdenich & Huber, 2002). Dominant behavior, for example displacement of a lower ranking subject, can have a negative impact on cooperation at two different stages: (1) the dominant may prevent the subordinate(s) from approaching or handling the apparatus containing a food reward that can be obtained by cooperation (e.g., Drea & Carter, 2009) and (2) the dominant may claim more than an even share of the reward after successful cooperation, demotivating the subordinate(s) to engage in subsequent cooperative interactions (e.g., Massen et al., 2015). Interestingly, the monopolization of the apparatus is infrequently measured in cooperation studies or is often physically impossible due to the separation of subjects. Nonetheless, work with hyenas in a cooperative task showed lack of displacements of subordinates from the apparatus dominants to be prerequisite to successful cooperation (Drea & Carter, 2009). Fruteau, Van Damme, and Noë (2013), in a coordination experiment with vervet monkeys, *Chlorocebus pygerythrus*, called this lack of displacements by dominants “showing restraint.” They showed that high-ranking animals were able to learn over time not to displace a low-ranking subject from a food container only she could open, leading to successful retrieval of rewards. Regarding the division of the reward in cooperation studies, it was found that dominants can also facilitate future cooperation by sharing the reward(s) more equally with subordinates (e.g., Massen et al., 2015; Schwing, Jocteur, Wein, Massen, & Noë, 2016).

Despite theoretical and practical evidence of the strong effect of social interactions during cooperation attempts, animals were often separated by walls or fences during cooperation tests (e.g., De Waal & Berger, 2000; Heaney, Gray, & Taylor, 2017; Mendres & de Waal, 2000; Schwing et al., 2016). While this was often done specifically to eliminate certain social factors and allow subjects to show their cognitive potential for cooperation (e.g., in kea; Schwing et al., 2016; Schwing et al., 2020), it may artificially facilitate cooperation by removing the need to abandon daily routines, such as displacing subordinates from resources or avoiding dominants. Partner control models of cooperation based on repeated games, such as the iterated prisoners’ dilemma, suggest, for example, that tolerance by dominants during the division of communally acquired rewards is important for successful future cooperation by the same individuals (Bshary & Noë, 2003). In many experimental set-ups the expression of dominance or tolerance is difficult or impossible because the animals are separated, the reward is indivisible, and/or the items or quantities each subject obtains are experimentally pre-determined. In addition to such immediate effects of behavior shown during cooperation attempts, the subjects’ social long-term relationships have been found to affect cooperation success too. In Barbary macaques, *Macaca sylvanus*, strong affiliation between subjects had a positive effect on cooperation (Molesti & Majolo, 2016). Similarly, in ravens higher affiliation was also found to lead to more cooperation success, although this was due to the animals’ acceptance of closely affiliated individuals in close proximity near the apparatus (Asakawa-Haas et al., 2016). Furthermore, rank distance, the difference in hierarchal position between subjects that is often used as a proxy for power differentials, was found to affect cooperation in chimpanzees (Suchak et al., 2014), with subjects closer in rank showing more cooperative success. However, as with affiliation in ravens, this effect was likely due to proximity effects, as subordinates were more reluctant to approach dominants the greater the rank distance was. Capuchins tested with an apparatus with a sliding tray baited with food that the subjects could reach by pulling bars also showed a proximity effect, though of a different nature (De Waal & Davis, 2003). Subjects cooperated better the further apart the rewards were placed, i.e., the more likely it became that the lower ranking subject would obtain at least some reward, with dominants allowing kin to obtain more than non-kin. Here proximity was thus also a factor, but during the reward-division phase rather than while approaching the apparatus. The dominant allowing the subordinate to approach the apparatus and/or taking part of the reward is therefore often crucial for successful cooperation.

Working with captive kea (*Nestor notabilis*), large parrots from New Zealand, we aimed to test which factors help or hinder cooperation among multiple animals that can freely interact with each other. We anticipated tolerance by dominant animals to be a major factor potentially impeding cooperation. We expected to see more tolerance, and hence more successful cooperation, among animals with stronger affiliative bonds and with smaller rank distances.

**Fig. 1:**
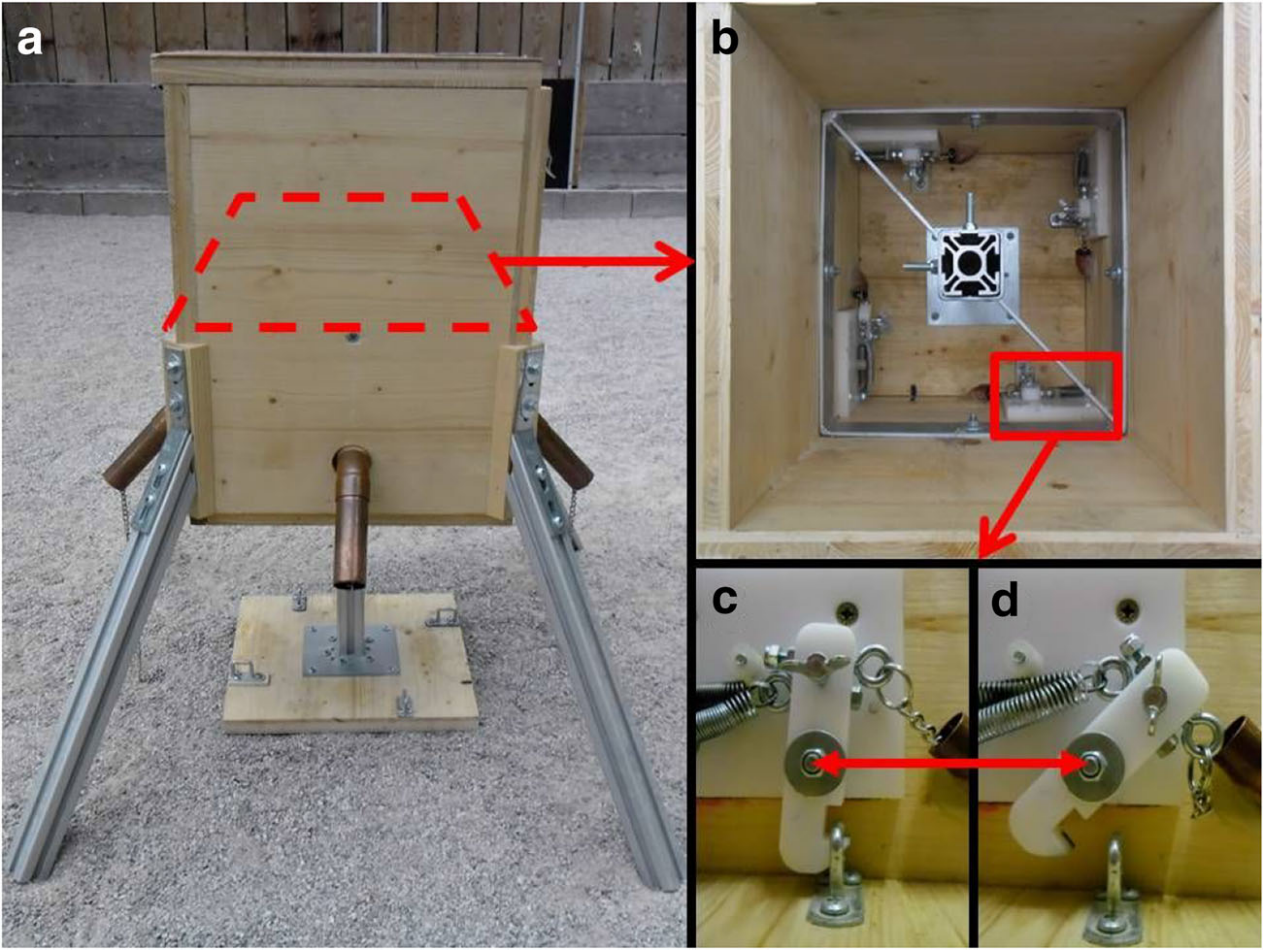
Four-chain box layout: (**A**) side-view of box with four chains and reward tray released, (**B**) top view of interior of box with locked tray, four locking mechanisms and central stabilizing bracket/tray guide, (**C**) detail-view of locking mechanism in locked position, (**D**) detail-view of locking mechanism in released position (this can be achieved by pulling on the attached chain or by tightening the wing screw for use of the box with less than four chains)

We started by familiarizing the animals with the apparatus individually by allowing them to pull a single chain that opened the lock holding the bottom of a wooden box. This allowed access to food rewards stuck on top of it. By adding a second chain to a lock at the opposite side of the box (see Fig. 1), we created a dyadic cooperation task in which two subjects had to pull two chains simultaneously to cause the baited bottom to drop. Additional chains could be attached to additional locks, such that three, or four birds, respectively, had to pull simultaneously to obtain rewards. The animals had to pull simultaneously, but they did not necessarily have to do so from the start, this is in contrast to tests based on the loose-string paradigm. This way we could test whether behavioral strategies that allowed success in the dyadic task would carry over to settings with the same apparatus under the same circumstances, but with three or four subjects. Tasks in which more than two animals can obtain rewards by acting in a coordinated fashion are rare (e.g., Fruteau et al., 2013; Suchak et al., 2014). This is surprising considering that many forms of cooperation in nature strongly depend on the behavior of multiple individuals, for example, in cooperative hunting by lions (Packer & Pusey, 1982; Stander, 1992) and other carnivores (Smith, Swanson, Reed, & Holekamp, 2012), as well as chimpanzees (Boesch, 1994), in cooperative defense of territories and other resources (Connor et al., 2017; De Weerd & Verbrugge, 2011; Farabaugh, Brown, & Hughes, 1992; Grinnell, 2002; Mares, Young, & Clutton-Brock, 2012; Radford & Fawcett, 2014) and in cooperative defense against predators (Arnold, 2000; Garay, 2009; Jungwirth, Josi, Walker, & Taborsky, 2015).

Kea (the singular and the plural are identical in the Maori language) are known to be curious and neophilic (Huber & Gajdon, 2006; Huber, Gajdon, Federspiel, & Werdenich, 2008). They are highly gregarious, yet social group compositions are frequently changing, with only the family unit, breeding pair and their offspring of the current year, representing a stable unit over time (Diamond & Bond, 1999). Although cooperative behavior to obtain food has not been observed in wild kea (a small fraction of rubbish bin-opening attempts did involve two birds acting simultaneously, but these were all unsuccessful; Gajdon, Fijn, & Huber, 2006), they can learn to exhibit tolerance in the presence of high-value food sources (carrion is a common food source in the wild, and while the adult birds were seemingly able to feed simultaneously, juveniles still exhibited aggressive behaviors in the presence of a thar carcass; Schwing, 2010). Importantly, kea are capable of dyadic cooperative behavior in the loose-string paradigm (Heaney et al., 2017; Schwing et al., 2016; Schwing et al., 2020). Many of their natural food items are extracted from the ground or from logs (Brejaart, 1994; Greer, Gajdon, & Nelson, 2015). The fact that they often allow conspecifics to forage in close proximity to such potential food sources suggests a propensity for restraint.

We expected high-ranking animals that had learned the value of being tolerant towards a single subordinate to show restraint in the presence of multiple subordinates too. We were especially interested in the behavior of middle-ranking animals in the three- and four-chain trials, since they had to induce tolerance by the highest-ranking animal, and at the same time tolerate the lower-ranking ones present. It turned out, however, that initially the dominant animals were so keen on defending the closed wooden box that none of them showed enough restraint to allow any subordinate present to handle the chains, even though we tested all possible dyads with the two-chain setup. We then decided to make monopolization as hard as possible by introducing the box with two chains attached with all 16 adult kea of our social group present. During this “group session” two birds managed to pull the chains simultaneously, after which multiple trials, ultimately involving all trained birds, resulted in the opening of the box during the same group-session. After this session, the high-ranking individuals permitted others to handle the chains and we could run our tests as originally planned, starting with two chains attached; thereafter we added a third and a fourth chain, respectively. We added extra birds in half of the two-chain and three-chain trials in order to make it harder for a single bird to control the whole apparatus, aiming to facilitate cooperation in a similar fashion to that of the group session.

This study was a pilot study in which we tested a new kind of apparatus and during which we proceeded from one phase to the next by trial and error. In doing so, we made some choices that, in hindsight, were not always optimal. Some of these choices prevented us from fully analyzing the data gathered in the experiments with two and three chains, as we explain in more detail below. We could use all the data collected in the main phase of the study in which four animals were required to obtain a shareable reward and enough from the preceding phases to identify the key steps that led to successful cooperation in this ultimate experiment.

## Materials and methods

### Subjects

Eight kea at the Haidlhof Research Station in Bad Vöslau (Lower Austria) participated as subjects in this study (see Online Supplemental Material (OSM), Table 1, for details). Another eight adults took part in a single “group session” during which all 16 members of our social group together had access to the apparatus in its two-chain configuration. All kea, 19 individuals in total, were kept in an outdoor aviary (52W × 10L × 4H m) with multiple compartments, furnished with branches, huts, feeding tables, enrichment areas and ponds. The experiments for this study were all conducted in a 6W x 10L x 4H m compartment that could be visually separated from the rest of the aviary. The kea were fed three times a day with fruit, vegetables, and seeds, and once daily with a protein source. Water was provided ad libitum.

**Table 1:** Number of sessions needed to reach criterion and advance from training to two-chain test; criterion was 8/10 correct in two consecutive sessions

	Jo	Fr	Pa	Ke	Ly	Wy	Pu	An
1st training	2	2	2	2	2	2	3	2
2nd training	2	7	2	2	7	3	2	3

### Experimental setup

**Fig. 2:**
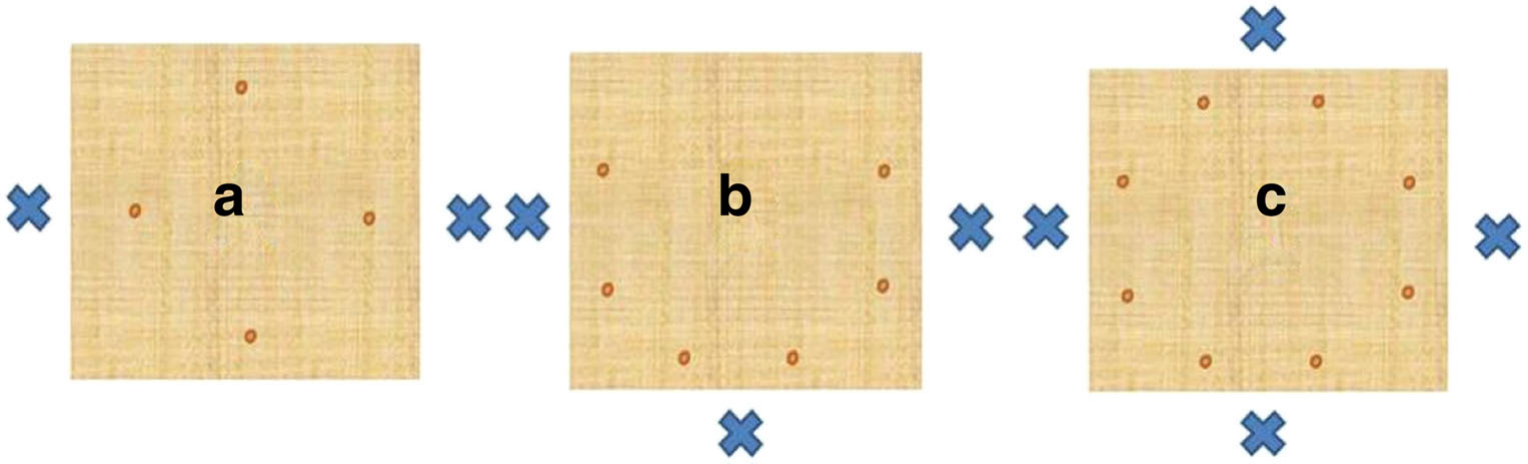
Reward placements (dots) on tray in relation to chain (crosses) placement in two-chain (**A**), three-chain (**B**), and four-chain (**C**) setups. Placements of the reward was chosen to allow asymmetric reward divisions, but the distance between food items prevented a single bird from eating all of them

The apparatus consisted of a wooden box (30W × 30L × 42H cm) with four steel legs (4W x 4L x 42H cm) attached at an angle at each corner to raise the box above the ground (Fig. 1A). The bottom of this box dropped down to the floor of the aviary when unlocked, exposing the food items, which consisted of small chunks of sticky “bird peanut butter” (CJ WildBird Foods Ltd), which remained in place. The birds got quickly used to the bottom dropping out without showing any signs of being startled or afraid. Up to four hooks, placed at each interior side of the box (Fig. 1B), could be used to lock the bottom in the closed position. Depending on the experimental setup, between one and four of the hooks were used (Fig. 1C); hooks not used were locked in an open position (Fig. 1D). Each hook in use was unlocked by pulling a chain attached to it, which protruded 30 cm out of the side of the box. Copper guide pipes (15 cm, 2.5 cm Ø, perpendicular to the side of the box, angled downward ~ 40°) for the chains were added after a pilot phase to improve the positioning of the subjects in relation to the dropping tray. The new positioning of the subjects required them to stand with their bodies oriented away from the apparatus, thus making it impossible for a pulling subject to stand directly underneath the bottom of the box. No bird was ever underneath the apparatus when the bottom dropped before or after installing the pipes. Additional birds present during some of the tests either attempted grabbing one of the chains or showed no interest in pulling and stayed away from the apparatus. Two pea-sized food chunks were added per chain in use. The configuration (see Fig. 2) allowed for higher ranking birds to obtain more than an even share but not to monopolize all rewards. In most cases participants were able to eat at least one item and a dominant bird could generally only take one or two nearby food items in addition. The tray was cleaned after each day of testing. One video camera captured kea behavior near the box and the reward division. A second wide-angle camera was used to capture the behaviors occurring between multiple birds. The training, two-chain, and three-chain sessions were conducted from 11 February until 22 May 2015 and the four-chain sessions from 1 September until 31 October 2015. All sessions were conducted between 09:00 and 12:00 or between 14:00 and 20:00, depending on daylight hours.

## Experimental procedures

### Individual training and general procedure

All subjects were individually isolated in the test compartment and allowed to explore the box with one chain in use. The criterion to proceed from the training to dyadic testing was success in at least eight out of ten trials in two consecutive sessions conducted on different days. Training of birds was conducted in two stages: a pilot phase was conducted over 3 days without the copper pipes, and the second phase after the copper pipes were installed lasted 9 days.

All trials started as soon as the box was set up with the bottom closed and ended either successfully with the bird(s) gaining access to the rewards by pulling all of the chains in use, or failed after one or more birds stopped interacting with the box/chains for 60 s. After each successful trial (except the tenth training session), the box was set up again in the presence of the subject(s). After a failed trial in training and in dyadic trails prior to the group session of 26 March 2015, the box was set up again and the trial repeated. After the group session any failed trial in the two-, three-, or four-chain setups ended a session.

### Two-chain setup – before group session of 26 March

Two birds were isolated from the group in the testing compartment, with the box with two chains required to be pulled to gain access to the rewards. After a total of 80 failed trials (ten dyads tested), subjects were given trials where upon first entering the testing compartment, the apparatus only had one chain installed. This allowed the higher-ranking subject to achieve success alone with a specific chain. The second chain was then added subsequently in the hope that the higher-ranking subject would continue guarding the first chain rather than monopolize the whole box. No successful cooperation was recorded in another 56 trials (ten dyads tested). See OSM for complete procedure.

### Two-chain setup in the group session

During the group session on 26 March 2015, the box with two chains in use was placed in the main compartment with 16 adult kea present: the eight subjects participating in the experiment and the remaining eight adults of our main group. Initially, the entire group (including juveniles) was present. However, as juvenile kea displace all other group members independent of size, age, or affiliation, all juveniles under 3 years of age were excluded after the second trial. Utilizing this setup, high-ranking birds could no longer defend the whole box but could still monopolize a single chain. This resulted in trials in which high-ranking individuals successfully opened the box together and ate the reward. In order to give the other subjects the same learning opportunities, we then started to remove birds one by one, starting with the highest ranking after 18 trials, then the second highest after another six trials, and so forth. After 40 successful trials (42 trials in total), each subject that was previously trained in single-chain sessions had successfully pulled a chain and opened the box at least once. The eight untrained adults, while displacing trained birds in the latter trials (after the four highest ranking trained birds had been removed), never attempted to pull a chain and were therefore not considered in the analysis.

The results from this session were analyzed to evaluate the effect on the post-group sessions with the two-chain setup as follows. The three different periods (pre-group sessions, group session, post-group sessions) were compared with one another in terms of the behavior seen around the box. The videos were coded for the number of displacements and the number of times each subject pulled the chains. In order to make comparison between the group session and dyadic post-group sessions possible, the number of displacements was adjusted for the number of birds present and the length of the trial (displacements per bird per minute). The number of displacements for each phase, further divided into successful and unsuccessful trials, was analyzed using a Kruskal-Wallis one-way ANOVA.

### Two-chain setup – post-group session condition

During a dyadic session, two to five trained subjects (see below for details) were isolated in the testing compartment with the box with two chains attached. Twenty-eight dyads were tested on their own at least once, while 26 of those pairings were tested with one or more additional birds present. In total 193 sessions were conducted, with differences in the motivation to participate causing some subjects to be present in more sessions than others (see OSM Table 6). The group ID or subject ID were used as factors in our models to avoid effects of pseudo-replication.

### Three-chain setup

Trials with three chains attached were conducted after all two-chain sessions had been completed. We tested 44 triads and a further 11 groups of four or five birds. In total 89 sessions were conducted, with differences in the motivation to participate causing some subjects to be present in more sessions than others.

### Test: Four-chain setup

The sessions with four chains were conducted after a 3-month break. During these sessions four birds were isolated in the testing compartment and no extra birds were ever added. We tested a total of 65 tetrads in 78 sessions.

## Analysis of factors influencing cooperation success

### Data coding

The presence of extra subjects in the two- and three-chain sessions after the group session resulted in different dyads and triads, respectively, opening the box during several of those sessions. While these were included to hinder monopolization, similar to the effect seen in the group session, the resulting data contained too much variation to allow for the analysis on session averages. Trial-by-trial analysis was also not possible due to the unbalanced nature of the data set in terms of successful and unsuccessful trials. We therefore decided only to use the data of the dyads and groups from the first trial of each two-chain session (N = 193) and the triads and groups in each three-chain session (N = 89). We compared these to the first trials of the four-chain sessions (N = 78) in a generalized linear mixed model (model 1, GLMM; Baayen, 2008).

The four-chain setup was only conducted with the required four subjects, which represented the setup with the greatest need for subjects to solve the task and was conducted when the birds had the greatest amount of experience with this type of setup. We therefore considered this the best representation of the factors limiting this type of cooperation, and thus also analyzed which factors influenced the rate of success within a session, with number of successful trials in a session as the measure of success (models 2a and b).

Displacements (one individual causing another to relocate) were counted for the phase before the subjects solved (or did not solve) the apparatus in each trial, which started when the apparatus was baited and closed by the experimenter and ended when the rewards were accessible or when one of the subjects refused to approach the apparatus any longer. In addition, the displacements by the highest-ranking subject were counted separately. Rank distance (rank based on displacements of subjects during the experiment) for each dyad of birds present was calculated. An affiliative score was calculated using the accumulative number of nearest neighbor events (birds found within 1 m of the subject) from the weekly group focal sampling (2 min focal sampling based on a lab-internal ethogram, approximately three samples a week per bird, period of data used: February 2013–May 2015).

Additionally, the number of rewards eaten by each subject was coded for each trial in the four-chain setup and the standard deviation of reward division was calculated as a measure of equal reward division amongst the birds, and thus tolerance in the presence of a divisible food source.

Inter-observer reliability of reward division, displacements by the highest-ranking subject, and displacements by all subjects were calculated by comparing with an external coder who was blind to the outcome of this study. The data compared comprised approximately 72% of the total data used in all analyses. While the data set was numerical, a strict categorical comparison was chosen to measure agreement per measure and trial. Perfect agreement resulted in a score of 1, with any discrepancy resulting in a 0. The percentage of perfect agreement for the different measures was: reward division 96.04%, displacements by highest ranking subject 97.91%, and displacements by all subjects 89.99% (see OSM for data set).

## Statistics

### Success in first trials in all setups (model 1)

To estimate to which extent the success in individual first trials per session correlated with a number of social factors, we fitted a GLMM (Baayen, 2008) with binomial error structure and logit link function (McCullagh & Nelder, 1989). As our key test predictors with fixed effect we included the maximum of the dyadic rank differences of the individuals present, the minimum of the dyadic affiliation indices of the individuals present, displacements by the highest-ranking subject, displacements by all subjects, and the number of birds present. We included trial type (factor with levels Dyadic, Triadic, and Tetradic) and the minimum number of successes the individuals present had witnessed or participated in during previous sessions to control for their effects too. As a random intercept effect, we included the particular group composition. We did not include any random slopes (Barr, Levy, Scheepers, & Tily, 2013; Schielzeth & Forstmeier, 2009) as most group compositions appeared at most a few times in the data, and, as a consequence, no random slopes component would have been identifiable.

As an overall test of the effect of the social predictors and to avoid “cryptic multiple testing” (Forstmeier & Schielzeth, 2011), we compared the full model with an otherwise identical null model lacking these predictors. We determined the significance of individual predictors by dropping them from the model one at a time and then comparing the fit of these reduced models with of the full model (Barr et al., 2013). All model comparisons were based on likelihood ratio tests (Dobson, 2002). Prior to fitting the model, we log-transformed (base *e*) the minimum dyadic affiliation score, displacements by the highest-ranking subject, displacements by all subjects, and the number of birds present to reduce the impact of influential cases. We also log-transformed the minimum of the numbers of successes per individual as we reasoned that the relevance of a given absolute increase would decrease with increasing number of successes. Prior to log-transforming, we added one to displacements by the highest-ranking subject, displacements by all subjects, and the minimum of the numbers of successes per individual. The sample analyzed with this model comprised a total of 359 trials (133 of which were successful) conducted with 133 different group compositions. Further considerations regarding the GLMMs are presented in the OSM.

### Number of successful trials in the tetradic condition (model 2a and b)

To estimate the extent to which the number of successful trials in sessions with the tetradic condition could be explained by social factors, we fitted a GLMM (model 2a) with negative binomial error distribution and log-link function. Into this we included maximum rank distance, minimum affiliation score, displacements by the highest-ranking subject, displacements by all subjects (for details and transformations see model 1) as test predictors and a random intercepts effect for the unique group composition. The null model comprised only the random intercepts effect, and the sample analyzed for this model comprised a total of 79 sessions conducted with 67 different group compositions. Overdispersion was not an issue (dispersion parameter: 0.708). Because of a relatively large number of sessions with zero successful trials (23 out of 78 sessions), we fitted an additional model complementing model 2a, which differed from the above described one in that it included an intercept for zero-inflation (function glmmTMB of the same package; version 1.0.0; Brooks et al., 2017).

The estimation of the effect of the division of the food obtained on the number of successful trials in a session was analyzed by fitting an additional model (model 2b) into which we included only sessions with at least one successful trial and which included the additional predictor SRP.rank_avg (the standard deviation of the rewards eaten by each subject, which was only defined for session with at least one successful trial). Apart from that, the model was identical to the one just described. The data for this model comprised a total of 56 sessions conducted with unique 48-group compositions. This model was also not over-dispersed (dispersion parameter: 1.039). Further considerations regarding the GLMMs are presented in the OSM.

## Results

### Individual training

We trained eight subjects to handle the apparatus with a single chain attached. All eight subjects passed the criterion of pulling and opening the box in eight out of ten trials on two consecutive days without the copper pipes (first training) in the minimum number of sessions, with the exception of Pu who needed one additional session. After the pipes were installed, a further training (second training) was conducted with the same criterion, as the apparatus had changed visibly. Four birds passed in the minimum number of sessions (Jo, Pa, Pu, Ke), with An and Wy needing one additional session and Fr and Ly five additional sessions to reach criterion (see Table 1). All birds showed perfect 10/10 success in the final two sessions of both first and second training.

## Towards cooperation by learning restraint

### Two-chain setup – phase 1

None of the dyads tested with the two-chain set-up managed to open the box during the first phase before the group session. The highest-ranking individuals present monopolized the box in most cases by chasing the subordinates away as soon as they approached one of the chains.

### Two-chain setup – group session

After identifying monopolization of the box as the main factor hindering cooperation, we arranged for a “group session” with the box in its two-chain setup and all adult individuals available present, i.e., our eight trained subjects and eight additional untrained birds. Initially the entire group was present, but juvenile kea can displace adults, including the trained birds, and were therefore removed soon after the session started (see below for details). We reasoned that any high-ranking individual would fail in his or her attempts to monopolize the whole box, and notably the two chains hanging at opposite sides, because they would be overwhelmed by the number of individuals to control. This arrangement worked very well. Two minutes into the session the box was opened for the first time by two middle-ranking individuals (Pa and Ke; see Table 2). After the box had been set up again, the juveniles (not subjects) started to monopolize the chains and were subsequently removed. Immediately following this, the same pair of subjects managed to open it for a second time. That apparently inspired the highest-ranking individual (Jo) to change his behavior. Rather than trying to defend the whole box when still closed, he took the place of Ke and opened the box with the help of Pa in the fourth trial. In the fifth trial the second-highest ranking bird (Fr) took Pa’s place and opened the box with Jo. This was followed by 13 trials in which Jo (rank 1) opened the box either with Fr (rank 2) or Pa (rank 3). We then removed Jo to give the rest of the group a better opportunity to handle the chains. This was followed by six trials in which Fr and Pa (rank 2 and 3) opened the box successfully. Between trials 24 and 25 we removed Fr and then Pa between trials 30 and 31. In the following trials the then highest-ranking animal (Ke – rank 4) continued to open the box assisted by Ly, Pu, or An. We removed Ke after trial 40, which led to only the second unsuccessful trial due to untrained birds that now also began to interact with the box. They did not do so in the presence of the four highest-ranking subjects, which were also amongst the highest-ranking birds in the group. We stopped the trial and reset, which gave Wy, the only subject who had not opened the box yet, the possibility to gain access to a chain and thus an opportunity to open the box together with Pu. Hence, by the time we reached the 40th successful trial all trained subjects were involved in a successful attempt at least once. The increasingly agitated behavior of the untrained birds led us to stop the session after this. The whole process is illustrated in Table 2.

**Table 2:**
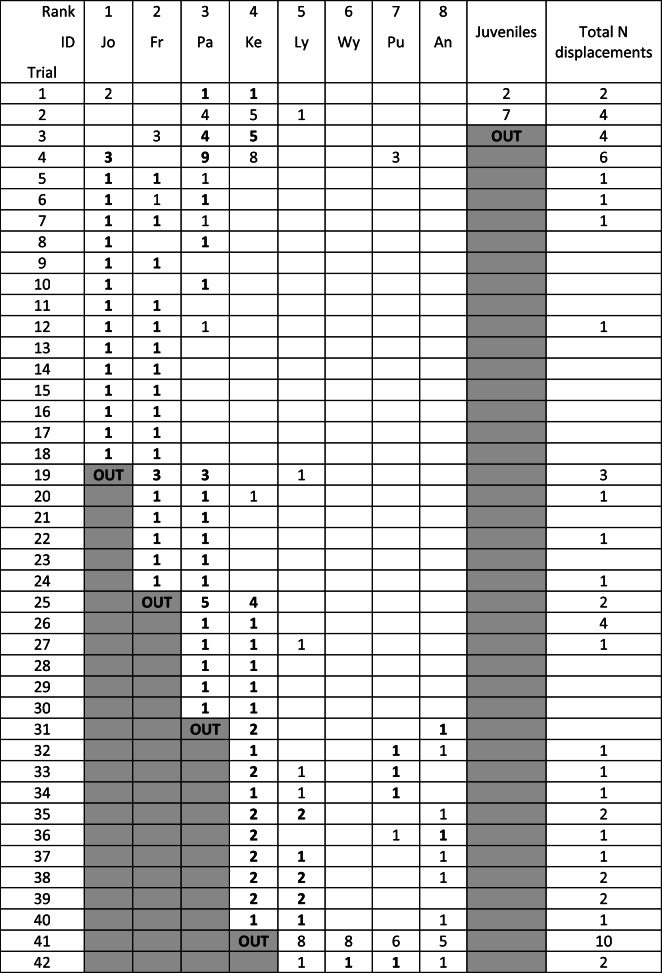
Illustration of the group session. x: the individual at the top of the column is involved in a successful attempt

OUT: the first trial in which the individual is no longer present. Light grey cells following “out” indicate the absence of the bird. Numbers in bird columns indicate number of chain pulls in a trial. Bold numbers indicate the bird being part of the successful dyad to solve the task. Total N displacements are all displacements between trained birds at the apparatus. Trials 2 and 41: in trial 2 the juveniles monopolized the apparatus, and were consequently removed from the group; in trial 41, the apparatus was repositioned after 5 minutes of constant displacements from both trained and untrained birds, which was hindering two birds pulling simultaneously, leading to almost immediate success with Wy and Pu

### Two-chain setup – phase 2

The trials with the two-chain setup and two to five birds present (see *Material and methods* for details) after the group session were often successful (Table 3). This could largely be explained by a change in the behavior of the dominant birds present, compared to the two-chain trials before the group session. They notably displaced the subordinates less when the latter approached one of the chains.

**Table 3:** Number of displacements during successful and failed two-chain trials before the group session (PRE), during the group session (GROUP), and after the group session (POST)

	Failure									Success					
	PRE			GROUP			POST			GROUP			POST		
	mean	SE	N	mean	SE	N	mean	SE	N	mean	SE	N	mean	SE	N
**Two subjects pulled chains**	1.31	0.17	35			0	1.33	0.73	9	1.08	0.21	40	0.13	0.03	494
**Total N trials**			152			3			138			40			494

We only show data from trials where two subjects attempted to solve the task by pulling chains. There were no successful attempts during the PRE-phase. Significant differences were found between the mean displacements of the PRE failure and the POST success phases (Dunn’s post hoc test with Bonferroni correction: H = 311.598, p < 0.001) and the POST failure and the POST success phases (Dunn’s post hoc test with Bonferroni correction: H = 160.390, p = 0.036)

## Cooperation by more than two kea

### Comparing the two-, three-, and four-chain trials

After the completion of the sessions with two chains attached to the box, we added the third chain, so that three birds had to pull simultaneously to open the box. We tested this setup with three to five birds present (see *Material and methods* for details).

After a break of several months, we tested the same eight subjects in groups of four with four chains attached to the box. All animals present therefore had to pull simultaneously to obtain the rewards.

The animals were slightly less successful in the three-chain than in the two-chain trials after the group session but reached a high success rate in the four-chain setup (Table 4).

**Table 4:** Success rate and displacements by all birds (adjusted for number of chains) of first trials in all sessions following the group session

	Two-chain	Three-chain	Four-chain
Number of sessions (first trials)	193	89	78
Success rate (%)	28.13	26.97	70.51
Displacements by all birds/number of chains	0.47	0.71	0.38

### Success in individual trials (model 1)

**Fig. 3:**
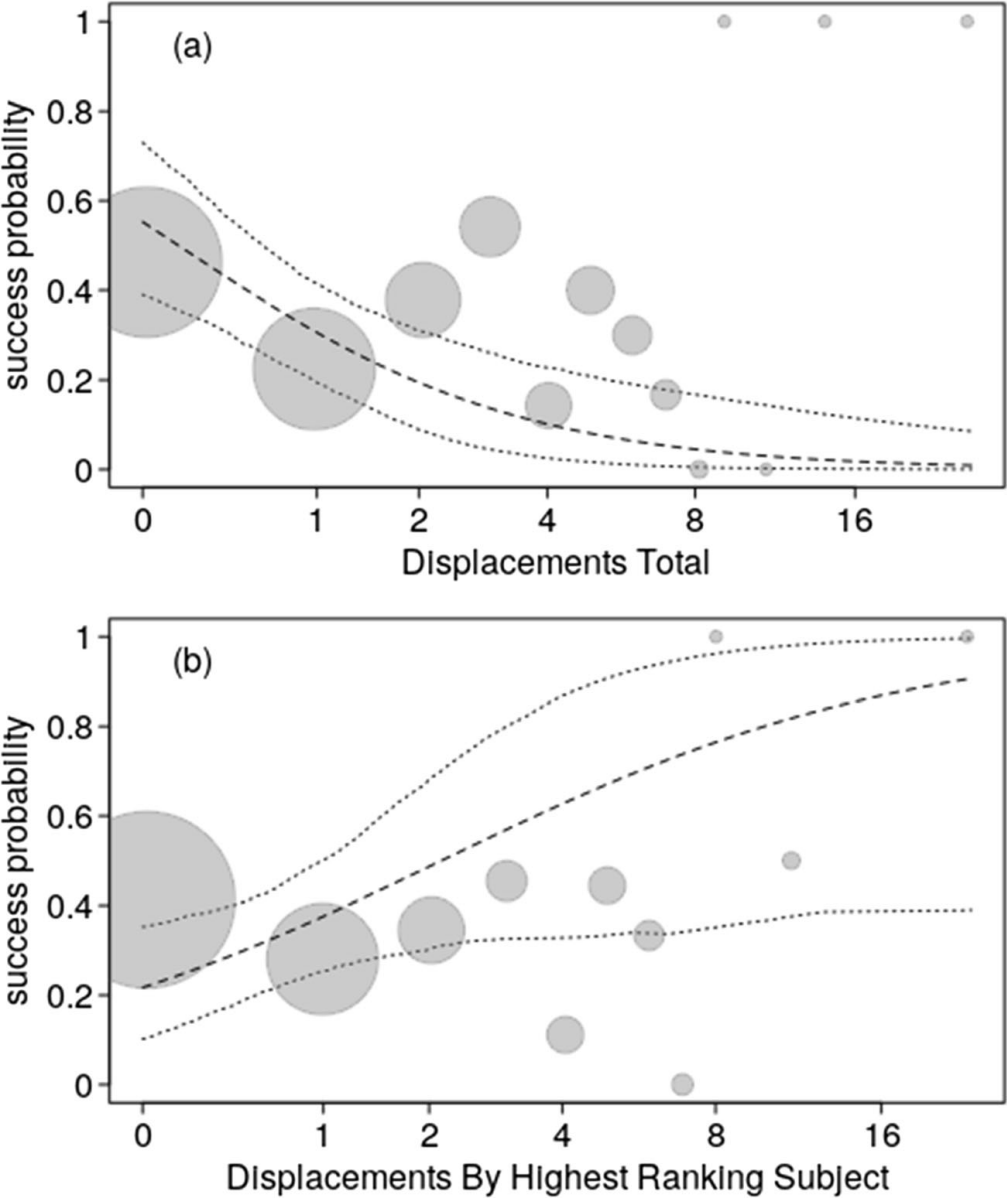
Probability of success in the first trial per session as a function of (**a**) displacements by all subjects and (**b**) displacements by the highest-ranking subject. Dots show the success probability (y-axis) per unique value of the predictor (x-axis), whereby the area of the dots depicts the number of trials per unique value of the predictor (range: 1–206). Dashed and dotted lines depict the fitted model and its confidence limits at all other predictors centered to a mean of zero

Overall, there was a clear influence of the test predictors on the probability of a success (full-null model comparison, likelihood ratio test: χ^2^ = 13.195, df = 5, p = 0.022). More specifically, we found that the more displacements occurred between all subjects the lower the probability of success in the first trial, while a weaker effect was found of higher numbers of displacements by the highest-ranking individual increasing the probability of success in first trials (Fig. 3; OSM Table 3). For the control predictors we found that trial type (two-, three-, or four-chain) had a significant effect on success probability whereas this was lowest for triadic and highest for tetradic trials (OSM Table 3). Furthermore, there was an effect of experience on success probability, with the number of successful trials previously experienced by the bird with the lowest number of successful trials amongst those present positively influencing the probability of success in the first trials.

### Number successful trials in the tetradic condition (model 2a and b)

**Fig. 4:**
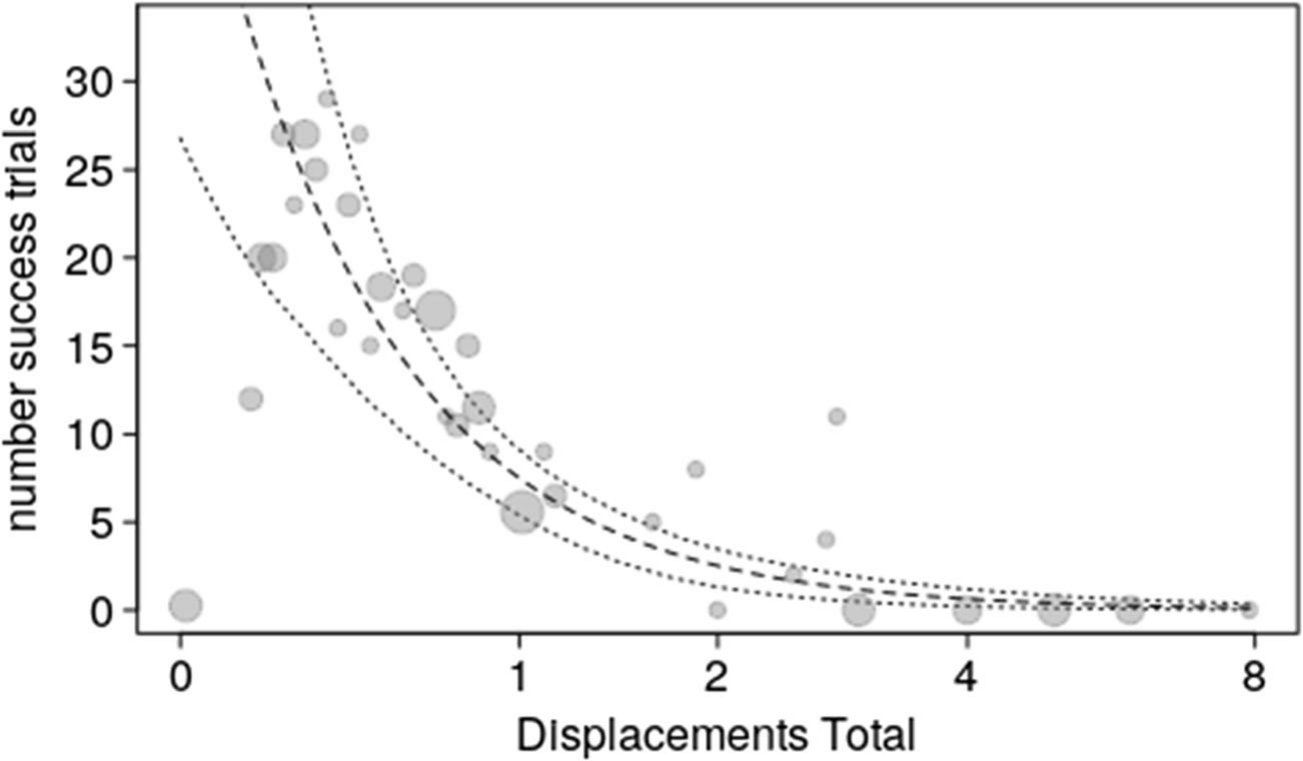
The number of successful trials per session as a function of displacements by all subjects. Dots show the actual observations whereby larger dots depict tied observations (range: 1–7 per value of AI before). The dashed and dotted lines depict the fitted model and its confidence limits at all other predictors centered to a mean of zero

For model 2a we also found a clear impact of the test predictors on the response (full-null model comparison: χ^2^ = 49.853, df = 4, p < 0.001). More specifically, the higher numbers of displacements by all subjects clearly decreased the number of successful trials in a session (OSM Table 4; Fig. 4). None of the other predictors had a significant effect on the number of successful trials per session. The model with zero-inflation revealed essentially identical results with the exception that it also revealed that the number of successful trials decreased with increasing maximum rank distance (Estimate ± SE = -0.199±0.089, χ^2^ = 5.095, df = 1, p = 0.024).

Model 2b showed basically the same result: a clearly significant full-null model comparison (c2 = 23.983, df = 5, p < 0.001). Similar to model 2a, it revealed that the number of successful trials decreased with increasing number of displacements by all subjects (OSM Table 5). None of the other predictors revealed significance, including reward division.

A further analysis, using the identities of the subjects rather than group IDs, was conducted and yielded results similar to those already found and were thus not followed up any further. A description of these can be found in the OSM.

## Discussion

Many factors can determine the difference between success and failure of cooperation in a setup such as we used in this study, with multiple partners all working on the same apparatus. We analyzed four in more detail because we considered them important based on both results of cooperation experiments with other species and our previous experience with kea: (1) monopolization of the apparatus, or essential parts thereof, by dominant animals, (2) reward division, (3) the power difference of subjects, as measured by rank distance, and (4) the affiliation qualities of their relationships. We summarize our results concerning each of these factors, before providing a comparison to the findings of similar studies with other species.

### The importance of restraint shown by dominants

The subjects learned that pulling a chain produced a food reward during the training with the box with a single chain attached. This meant that they learned the association between the apparatus and food. It is not surprising that animals attempt to defend an object that is known to contain food. It turned out that the higher-ranking kea were displacing the other subject present in the trials to prevent the subordinates from access to both chains in spite of a considerable distance between the two chains. This monopolization of the apparatus turned out to be a major hurdle preventing any spontaneous success in the first round of sessions with two chains.

That hurdle was overcome during a “group session” in which so many animals were present that even the highest-ranking animal (Jo) did not manage to control the apparatus completely. Jo attempted to do so, nevertheless, and while he was busy doing so, two other animals managed to grab a chain each and opened the box (see Table 2 for details). A few trials later Jo pulled a chain himself and thus experienced the benefits of cooperation. This way the most dominant bird learned, gradually or instantly, that some restraint in his behavior towards his group members yielded a positive result. We then made sure that all animals that were dominant over at least one other subject could have the same learning experience by taking out the highest-ranking birds one by one, until all trained birds had gained access to the rewards by pulling a chain at least once. This result – animals learning restraint one by one from high to low in a dominance hierarchy – is strongly reminiscent of the results of a study with vervets (Fruteau et al., 2013), except that none of the vervets were physically removed and lower-ranking ones therefore learned to restrain themselves in the presence of higher-ranking animals. Our results corroborate the findings of only a handful studies (Drea & Carter, 2009; Fruteau et al., 2013) that showed that dominants have to learn restraint before any successful cooperation is possible. This apparently is a very difficult, but not impossible, thing to learn. In fact, our subjects further down the hierarchy learned it too in spite of having very few opportunities with first-hand experience. It is very possible, however, that they learned by observing the actions of the higher-ranking animals in earlier trials during the group session too. When dominant members of social groups do not show restraint in comparable situations, be it in captivity or in nature, cooperation becomes impossible and the influence of other factors, such as reward division, power differentials, and affiliation, have little to no effect.

### Is “learning restraint” instantaneous or gradual?

The rather sudden switch the higher-ranking animals made in their behavioral strategy during the group session suggests instantaneous learning, as if they had gained an “insight” into the important role partners played in obtaining rewards. It is, however, very possible that they also learned certain aspects gradually over the course of the experiment during which they were confronted with basically the same apparatus over more than 1,500 trials. It is also possible that not only did the high-ranking animals learned to restrain themselves, but the low-ranking animals also learned to act more persistently. Future research including naïve kea as either the highest- or lowest-ranking subjects might provide results to better explain the exact influence of restraint and persistence on cooperative success.

We indeed found a gradual learning effect at the group level by analyzing the probability of success in first trials across all setups. The success rate increased with the number of successful trials in which the least experienced subject involved had taken part. While this result suggests an order effect with a gradually increasing success rate over the sessions in spite of adding extra chains, we found in fact that the success rate in the three-chain session dropped compared to the two-chain sessions in the preceding phase of the experiment and reached its highest level in the four-chain setup (Table 4). How can this be explained? Looking at the data, one finds a few trials in the first sessions after adding the third chain with displacement frequencies by the highest-ranking subject far above average, which significantly contributed to the overall low success rate in the three-chain sessions. One factor might have been the arrangement of the chains (see Fig. 2). All birds now had a direct neighbor rather than only one partner on the opposite side of the apparatus. Standing at the chain in the middle, a dominant animal now also had the two other chains in striking distance, allowing it to defend all three chains. The defense of all chains was practically impossible in the four-chain setup. We cannot draw any strong conclusions about the exact nature of the learning process(es), however, not least because we made the task more complicated by adding chains, while at the same time the subjects gained in experience.

### Does resource division play a role?

The reward was offered in such a way that the default reward division was two items for each participant (see Fig. 2), but an animal that ate fast enough and was dominant enough could in addition eat one or two items (in very rare occasions even three) placed under a chain she/he did not pull. The data show that the rewards were indeed often asymmetrically divided and often enough an animal that assisted in opening the box did not obtain any reward, while others received more than their fair share (see OSM Table 6).

We could not find any clear sign that a disadvantageous resource division had any negative effect on the eagerness of animals to participate in more trials within a session. A closer look at the data showed that some of the animals remained motivated to participate even after trials in which they contributed to the opening of the box but got no reward at all. This might seem puzzling when one looks at the results with classical game-theoretical “partner control” models of cooperation in mind (Bshary & Noë, 2003), but that might not be the right way to interpret the kea’s behavior. We do not know whether the kea experience our experimental setup as a social problem that needs to be solved by interacting with group members or a technical problem where the rewards are unpredictable. We defined “successful cooperation” here as an outcome of a trial in which the box was opened, but we cannot show that the kea “cooperated” in the social sense of the term, meaning that they paid attention to and coordinated with the movements of their partners. It might well be that the kea experienced our box as if it were a one-armed (or “one-chained”) bandit that occasionally yields a reward when a chain is pulled (Noë, 2006). While we cannot say with any certainty that they understood how the other birds’ actions played a role in opening the box, the change in behavior towards the other birds suggests that they did learn a need for them to have access to the apparatus to gain access to the rewards.

The more dominant participants’ trials often got more than their “fair” share (see OSM Table 7) of successful attempts, i.e., more than the two items. This will in all likelihood have motivated these birds to continue with chain pulling. It may also have contributed to their willingness to show restraint, but we would need separate and rather elaborate experiments to prove that.

### The effect of rank distance

The more powerful the highest-ranking animal present is, the easier it is for him or her to monopolize the apparatus and hence prevent successful cooperation. We quantified power differentials simply as rank distances and notably looked at the relationship between the greatest rank distance of any two subjects present in a session and the outcome of the trials. In many dyadic trials and all triadic and tetradic trials, there were more than two individuals present. We chose the greatest rank distance as the simplest and most relevant parameter we could use, but we are aware of the fact that power differentials among multiple subjects may have more complex effects than this simple variable can reveal.

We found that the lower the maximum rank distance was the higher the proportion of successful trials in a session. A similar effect was found in chimpanzees (Suchak et al., 2014), where it was shown that decreasing rank distance improved cooperation success. However, the opposite effect was found in ravens (Massen et al., 2015), where the authors suggested that competition between individuals decreased with increasing rank distance, leading to greater success. Previous work on macaques has suggested differences in the strength of a species-typical hierarchal organization can explain differences in the tolerance of members of different hierarchal positions (Petit et al., 1992). Unlike the primate species tested on cooperation, e.g. macaques, kea have non-linear hierarchies with great seasonal fluctuations (Diamond & Bond, 1999; Jackson, 1960; Tebbich, Taborsky, & Winkler, 1996). In a “seesaw” experiment with kea performed by Tebbich et al. (1996), a greater power differential made it more likely for the dominant to obtain a reward, because it made it easier to coerce the subordinate to fulfil its task-specific role. Harassment of partners therefore helped the dominant subject to obtain a reward, while in our experiment harassment by the dominant made obtaining a reward less likely. Most successful trials in our experiment fulfil the instrumental definition of cooperation as “an outcome attained through coordinated action and benefit to all participants” in all instances in which all subjects that pulled a chain got a reward, while the seesaw experiment does not qualify as cooperation according to any generally accepted definition (Noë, 2006), as the outcome was often not beneficial for the subordinate. It should be noted, however, that over the course of the experiment kea in both studies benefitted from their actions by gaining rewards, but also experienced trials in which they got no reward for their actions; so while the coercion of subjects to act without benefit within a trial would not be considered cooperation, previous experience of getting a reward could have motivated subjects in both studies to “cooperate.”

### No obvious effect of relationship quality

In contrast to other studies on cooperation, we found no effect of affiliation, as was the case in primates and corvids (Asakawa-Haas et al., 2016; Molesti & Majolo, 2016) but also in kea when tested with physical separations (Schwing et al., 2016). In ravens, an indirect effect of affiliation effect was found: proximity to the partner was found to be the underlying factor, with affiliation facilitating the proximity needed for successful cooperation. Once the proximity constraint was removed, affiliation was no longer a significant factor (Asakawa-Haas et al., 2016). In kea, previous cooperation work showed effects of strong affiliation on cooperation success despite the physical separation of the subjects (Schwing et al., 2016), while here, with no separation, there were also no effects of affiliation. However, a follow-up study to the 2016 loose-string paradigm experiment implemented a training that caused the subjects to pay closer attention to the action of a human partner (Schwing et al., 2020). The resulting increase in success rate for all subjects when compared with the previous experiment suggests that the effect of affiliation found in the initial study was due to more affiliated birds being on the apparatus together at the correct time, and thus an effect of proximity despite the physical separation. Once the subjects learned to pay attention to the actions of the partner, a key piece of information regarding how the cooperative actions works, this effect of affiliation disappeared. That training likely played a similar role to the group session in this study, in that the subjects learned a cause-effect relationship between their actions and the outcome.

There is an inherent problem with the representation of affiliations in multi-subject groups. Any individual required to coordinate with the others can disrupt success, therefore we chose to include the minimum affiliation of any dyad present in a group to be able to compare across groups. Similar to the rank distance, the actual effect of any pair’s affiliation on the cooperation success as a whole is likely much more complex. Future studies, where only such parameters are varied, would be necessary to investigate the true nature of multi-subject affiliative effects on cooperation.

### How do our results in dyadic cooperation compare to those of previous studies?

We trained our subjects to make them familiar with the apparatus, as is customary in these sort of study. Kea are neophilic and generally highly interested in object manipulation, so it was not surprising to see that they quickly learned to open the box with a single chain attached. Our subjects did not solve the dyadic tasks spontaneously, as the dominant animals were defending the apparatus as a valued resource, as discussed above. We chose to present the high-ranking individuals with a group setting where the number of individuals the apparatus had to be defended from was too great to be achieved. After the high-ranking animals had learned to show more restraint, our kea performed rather well in the dyadic task, both with two and with more than two subjects present (see Table 3). The results suggest that it was not a mere overall decrease in displacements, however, as failed two-chain trials after the group session still showed displacement frequencies comparable to the trials before the group session. It is also unlikely that success was due to more persistent behavior of lower-ranking subjects, because after the group session we did not increase attempts by subordinates to approach and handle chains after being displaced by a dominant animal.

It is difficult to compare our results with those of studies in which animals are physically separated from each other while performing cooperative tasks, even if only by an immediate partition (Scheid & Noë, 2010; Stephens, McLinn, & Stevens, 2002), because such studies lack the requirement of dominants’ behavioral adjustments towards subordinate partners to obtain rewards. To highlight this difficulty, we would like to compare two well-known studies with subjects belonging to the same species, capuchin monkeys, but that differed – among other things – in the degree to which the subjects could interact with each other. In a study by Chalmeau, Lardeux, Brandibas, and Gallo (1997) the subjects had to push two levers simultaneously that were 60 cm apart, too far for a single monkey to handle. The authors’ main conclusion was that the capuchins did not grasp the importance of the role played by the partner. In a study by Mendres and De Waal (2000), two monkeys had to pull a platform towards their cages by each pulling a handlebar. In contrast to the previously mentioned study, their subjects showed insight in the effect of the partner’s behavior on the outcome of the trials. Mendres and De Waal attributed this discrepancy to the difference in the complexity of the apparatus used. They reasoned that their subjects could directly see the effect of their partner’s behavior, while those in the study by Chalmeau and colleagues could not. That may indeed largely explain the differences, but another aspect was overlooked: in the Chalmeau study the subjects were members of a small group that could all reach the levers and, more importantly, interfere with group members that attempted to do so, while the capuchins in the Mendres and De Waal study sat in separate cages. In both experiments, like in ours, the animals could still bring the task to a successful end by handling the apparatus simultaneously even after one animal pushed the lever or pulled the handle alone. This aspect makes our results, and the results of the studies just mentioned and similar ones, hard to compare to studies in which the subjects are explicitly prevented from solving the task when one of the subjects acts without waiting for the partner. Such tasks are notably designed to show whether or not animals appreciate the role the partner plays in the outcome of the task. The best examples of the latter type of dyadic cooperation experiments are those based on Hirata’s “loose-string” paradigm (Hirata, 2003), which have now been done with a fair number of different species, for example chimpanzees (Hirata & Fuwa, 2007; Melis et al., 2006), rooks (Seed et al., 2008), elephants (Plotnik et al., 2011), ravens (Asakawa-Haas et al., 2016; Massen et al., 2015), and a string of other species, among which are kea (Heaney et al., 2017; Schwing et al., 2016; Schwing et al., 2020). A serious constraint of the loose-string paradigm, and a reason for us not to use it, is that it is very hard to scale it up for use by more than two animals simultaneously. The price we pay, however, is that our experiment conveys little information about the kea’s insight into the role of the partner. However, we could show in another experiment with the same subjects that kea indeed understand the need for a partner in a loose string dyadic test (Schwing et al., 2020).

### Is cooperation by multiple subjects inherently more complex than dyadic cooperation?

The success rate in the three-chain sessions was a bit lower than in the preceding two-chain sessions but was very high again in the four-chain session that we performed after a break of a few months. It seems therefore that the step from coordinating with a single partner to two or three was not very hard to take by our subjects. This may not be too surprising, because we did not make n-agent cooperation as hard for them as we could have done. A precocious action of a single individual could not make the task unsolvable, as in a dyadic loose-string task. Our animals could start pulling and hope for others to start pulling too. Technically, it would have been possible to have the release mechanism of the bottom of the box block if the required number of subjects would not synchronize from the start, but such a complicated mechanism would have given our animals little chance to learn which action has which effect. The locking mechanism we used was hidden from view too, but the effect of pulling one or more chains was pretty straightforward. We could also have made the resource shareable but easily monopolizable. This probably would have made the changes of obtaining a reward too low for lower-ranking participants to keep them motivated to participate.

### How does our experiment compare to some of the few experiments that required n-agent cooperation published to date?

#### Hyenas

Drea and Carter (2009) tested captive spotted hyenas in a cooperative task using a setup similar to our two chain setup: a collapsible platform that required two ropes to be pulled simultaneously to allow a food reward to drop. Also similar to our methodology, the hyenas were given experience with the apparatus in training trials requiring only one rope to be pulled. Furthermore, they also conducted trials with four individuals, but only two were needed to solve the task. While they did also test a setup where all four animals could act as subjects, this was done by presenting the hyenas with two setups, rather than a single one where all four needed to work together. Nonetheless, in terms of similarity, this is the most comparable to our box. Similar to the kea, the hyenas solved the training quite quickly. However, they then also solved the dyadic setup very quickly, suggesting that the dominant individual already tolerated the lower ranking subject in the vicinity of the apparatus. As the apparatus was a collapsible platform above, rather than a box which could be inspected from all sides, it could be that the setup was not seen to the same degree as a monopolizable object; however, the ropes would likely still have been associated with the food reward, and thus the dominant hyenas showed more restraint than the dominant kea. Interestingly, the addition of animals improved success overall, albeit with the rank related aggression between partners impairing performance.

#### Vervets

As part of an investigation of market effects in two free-ranging group and one captive group of vervets, Fruteau, Voelkl, Van Damme, and Noë (2009); Fruteau et al. (2013) trained low-ranking adult females to open containers with a rich food source that could be shared with their entire group. The closed containers with the food source visible could be monopolized by the high-ranking members of the group, however, and they could eat the lion’s share of the food once the containers were opened. The trained females would therefore not open the containers with any of the group members ranking above her (three, five, and eight, respectively, depending on the group) anywhere near to container. This created a complex social dilemma in which all animals dominant to the female had to learn to all stay outside an imaginary “forbidden circle” with a radius of roughly 10 m simultaneously, since otherwise the female would refuse to open the container. During this experiment, which inspired the present kea study, all dominants learned to restrain themselves and stay outside the forbidden circle one by one from high to low in the rank order. The vervets could learn by correctly “reading” the reactions of the trained female to their own actions and might, or might not, have learned socially by observing the interaction between the female and other dominants. The high-ranking kea in our study had to learn how their actions caused more, or less, desirable behavior in their subordinates and they too might have learned, or not, from the interaction between other dyads.

#### Chimpanzees

Suchak et al. (2014, 2016) used an apparatus that required two or three members of a captive chimpanzee group to pull in a tray with food. The members of the group had free choice of whether and with whom to participate. Unlike our experiment, the actions required to obtain the reward were different for the participants. One animal (dyadic condition) or two animals (triadic condition) had to remove a barrier, allowing the third to pull the tray. The food obtained dropped directly in front of the animals participating and could easily be shielded from the other participants and non-participating members of the group. As in our experiment, the chimpanzees could, and did, handle the apparatus in the absence of partners without blocking the apparatus. Ten of the 11 members of a well-established group participated successfully in this task, which was solved without specific training. Success rates were lower in a newly formed group of 15 chimpanzees, but still remarkably high. There was a preference for working with close kin and with animals close in rank in the well-established group. We too found that less difference in rank greased the wheels of cooperation. Remarkable in this study is that, in spite of the steep rank-order chimpanzees are known to have, the dominants did not monopolize the apparatus and there were relatively few attempts to steal the food items produced. The amount of competitive behavior dropped in favor of more cooperative behavior over the course of the thousands of trials conducted in this study.

## Conclusions

We showed that there is an overwhelmingly important factor that determines the success of failure of solving this type of cooperation task in kea: the highest-ranking animal present must learn to give up on monopolizing the whole apparatus and allow the subordinate(s) to approach and participate. Once such restraint has been learned by dominants, solving the task turned out to be relatively easy, albeit still strongly affected by displacements of subordinates by dominants. This was true even after we made it harder by requiring more individuals to act simultaneously in order to obtain food rewards. Other factors, such as a small difference in rank and previous experience with success contributed to solving the task successfully too, while affiliation did not, but those effects became visible only after the hurdle of “learning restraint” had been taken. While there are many factors that would still need further study to determine their effect on cooperation, we were able to show for the first time that four kea can work simultaneously on the same apparatus to gain access to a sharable reward.

## Supplementary Information

ESM 1 (DOCX 43 kb)

ESM 2 (MP4 9683 kb)

ESM 3 (MP4 9949 kb)

ESM 4 (MP4 11073 kb)

ESM 5 (XLSX 67 kb)

ESM 6 (XLSX 201 kb)
