# Supplementary material for: Kea, Nestor notabilis, achieve cooperation in dyads, triads, and tetrads when dominants show restraint
Source: Learn Behav. 2021 Feb 2;49(1):36–53. doi: 10.3758/s13420-021-00462-9 (PMC7979628; doi:10.3758/s13420-021-00462-9)
Supplement: Supplementary file 1 — (DOCX 43 kb) [file 13420_2021_462_MOESM1_ESM.docx]

# Supplementary Information

SI Table 1 Subject information

| Name/abbreviation | abbreviation | Age (years) | age group | Sex |
| --- | --- | --- | --- | --- |
| John | Jo | 16 | adult | m |
| Frowin | Fr | 11 | adult | m |
| Paul | Pa | 5 | adult | m |
| Kermit | Ke | 11 | adult | m |
| Lily | Ly | 8 | adult | f |
| Willy | Wy | 8 | adult | f |
| Papu | Pu | 3 | sub-adult | f |
| Anu | An | 8 | adult | m |

SI Table 2 Frequency of participation for each subject in different groups sizes for the two-chain setup.

| number of birds | Jo | Fr | Pa | Ke | Ly | Wy | Pu | An |
| --- | --- | --- | --- | --- | --- | --- | --- | --- |
| 2 | 7 | 7 | 7 | 7 | 7 | 7 | 7 | 7 |
| 3 | 5 | 6 | 6 | 7 | 8 | 6 | 5 | 3 |
| 4 | 4 | 1 | 3 | 1 | 4 | 3 | 4 | 3 |
| 5 | 3 | 1 | 2 | 1 | 4 | 4 | 4 | 5 |

## Additional remarks on the pre group session testing

No successful two-chain cooperation was recorded in the first attempts (40 trials, 10 dyads). After installing the copper pipes and providing additional individual training (40 trials, 10 dyads) there was no improvement. Next, the subjects were first confronted with a box with one chain in use, to which a second chain was added in plain view of the subjects, after the dominant had solved the one-chain box for several trials in a row; this was done to highlight the addition of the chain. This also led to only negative results (56 trials, 10 dyads).

## General considerations for the GLMMs

We fitted the models in R (version 3.6.3; (Team, 2020)) using the functions glmer (model 1) or glmer.nb (model 2a) of the package lme4 (version 1.1-21; (Bates, Mächler, Bolker, & Walker, 2015)) or the function glmmTMB of the equally named package (version 1.0.0; (Brooks et al., 2017)). Prior to fitting the models, we z-transformed all quantitative predictors to a mean of zero and a standard deviation of one to ease model convergence. We estimated model stability by dropping group compositions one at a time from the data and comparing the estimates obtained from models fitted to these subsets with those obtained for the full data set, which revealed both models to be of good stability (see results). We determined confidence intervals of model estimates and fitted values by means of parametric bootstraps (N=1,000 bootstraps; function bootMer of the package lme4). Collinearity was no issue in either of the two models (model 1: maximum of squared Generalized Variance Inflation Factors after having taken them to the power of 1/twice their degrees of freedom = 2.968; maximum Variance Inflation Factor models 2a and b: 2.11; estimated for a respective standard linear model lacking the random effect; (Field, 2005; Fox & Monette, 1992)). Lastly, inclusion of unique group composition was chosen over the presence of specific individuals in the main analysis, as the specific dynamic of each subgroup was estimated to have a greater effect on the outcome of any trial/session, than the inclusion of any specific subject. Nonetheless, the models were also run with individual ID rather than the group composition, to check for variation in the outcome (see Section: Further analysis of the effect of individual subjects).

SI Table 3. Results of model 1 with the probability of success in individual first trials per session as the response (estimates together with standard errors, confidence limits, significance tests, as well as minimum and maximum of model estimates obtained after dropping unique group compositions one at a time). DHRS: displacements by highest ranking subject, DAS: displacements by all subjects.

| term | Estimate | SE | lower Cl | upper Cl | χ^2^ | df | P | min | max |
| --- | --- | --- | --- | --- | --- | --- | --- | --- | --- |
| intercept | -0.839 | 0.385 | -1.664 | -0.123 |  |  | ^(1)^ | -1.017 | -0.633 |
| max. rank dist.^(2)^ | 0.359 | 0.300 | -0.246 | 0.918 | 1.537 | 1 | 0.215 | 0.263 | 0.459 |
| min. aff.^(3)^ | 0.218 | 0.296 | -0.373 | 0.834 | 0.550 | 1 | 0.458 | 0.130 | 0.343 |
| DHRS^(4)^ | 0.693 | 0.335 | 0.082 | 1.428 | 4.645 | 1 | 0.031 | 0.610 | 0.986 |
| DAS^(5)^ | -1.016 | 0.355 | -1.821 | -0.460 | 9.466 | 1 | 0.002 | -1.304 | -0.901 |
| nr. birds present^(6)^ | 0.345 | 0.375 | -0.403 | 1.097 | 0.923 | 1 | 0.337 | 0.123 | 0.518 |
| TypeTriadic^(7)^ | -0.908 | 0.625 | -2.145 | 0.137 | 13.795 | 2 | 0.001 | -1.185 | -0.675 |
| TypeTetradic^(7)^ | 1.327 | 0.794 | -0.192 | 3.043 |  |  |  | 0.909 | 1.658 |
| min. nr. succ.^(8)^ | 0.860 | 0.332 | 0.320 | 1.462 | 9.109 | 1 | 0.003 | 0.720 | 1.020 |

^(1)^ not indicated because of having a very limited interpretation
^(2)^ z-transformed to a mean of zero and a standard deviation (sd) of one; mean and sd of the original variable were 4.003 and 1.908, respectively
^(3)^ log- and then z-transformed; mean and sd of the original log-transformed variable were 2.075 and 0.955, respectively
^(4)^ log- and then z-transformed; mean and sd of the original log-transformed variable (+1) were 0.454 and 0.618, respectively
^(5)^ log- and then z-transformed; mean and sd of the original log-transformed variable (+1) were 0.664 and 0.683, respectively
^(6)^ z-transformed; mean and sd of the original variable were 3.237 and 0.970, respectively
^(7)^ dummy coded with dyadic being the reference category; the indicated test refers to the overall effect of the factor
^(8)^ log- and then z-transformed; mean and sd of the original log-transformed variable (+1) were 3.981 and 1.871, respectively

SI table 4. Results of model 2a with the number of successful trials per session as the response (estimates together with standard errors, confidence limits, significance tests, as well as minimum and maximum of model estimates obtained after dropping unique group compositions one at a time). DHRS: displacements by highest ranking subject, DAS: displacements by all subjects.

| term | Estimate | SE | lower Cl | upper Cl | χ^2^ | df | P | min | max |
| --- | --- | --- | --- | --- | --- | --- | --- | --- | --- |
| intercept | 1.946 | 0.125 | 1.604 | 2.143 |  |  | ^(1)^ | 1.582 | 1.983 |
| max. rank dist.^(2)^ | -0.188 | 0.130 | -0.443 | 0.067 | 2.126 | 1 | 0.145 | -0.218 | -0.014 |
| min. aff.^(3)^ | 0.000 | 0.129 | -0.258 | 0.258 | 0.000 | 1 | 0.998 | -0.099 | 0.033 |
| DHRS^(4)^ | 0.042 | 0.281 | -0.453 | 0.547 | 0.022 | 1 | 0.882 | -0.280 | 0.211 |
| DAS^(5)^ | -1.518 | 0.276 | -2.070 | -1.143 | 31.206 | 1 | 0.000 | -1.769 | -1.374 |

^(1)^ not indicated because of having a very limited interpretation, respectively

^(2)^ z-transformed to a mean of zero and a standard deviation (sd) of one; mean and sd of the original variable were 5.367 and 1.200, respectively

^(3)^ log- and then z-transformed; mean and sd of the original log-transformed variable were 1.459 and 0.584, respectively

^(4)^ log- and then z-transformed; mean and sd of the original log-transformed variable (+1) were 0.343 and 0.481, respectively

^(5)^ log- and then z-transformed; mean and sd of the original log-transformed variable (+1) were 0.719 and 0.564

^(6)^ log- and then z-transformed; mean and sd of the original log-transformed variable (+1) were 0.730 and 0.576

SI table 5. Results of model 2b with the number of successful trials per session as the response and including SRP.rank_avg as an additional predictor (estimates together with standard errors, confidence limits, significance tests, as well as minimum and maximum of model estimates obtained after dropping unique group compositions one at a time). DHRS: displacements by highest ranking subject, DAS: displacements by all subjects.

| term | Estimate | SE | lower Cl | upper Cl | c2 | df | P | min | max |
| --- | --- | --- | --- | --- | --- | --- | --- | --- | --- |
| intercept | 2.715 | 0.068 | 2.523 | 2.817 |  |  | ^(1)^ | 2.686 | 2.733 |
| max. rank dist.^(2)^ | -0.079 | 0.078 | -0.225 | 0.073 | 1.037 | 1 | 0.309 | -0.121 | -0.051 |
| min. aff.^(3)^ | -0.053 | 0.073 | -0.206 | 0.091 | 0.528 | 1 | 0.467 | -0.093 | -0.026 |
| DHRS^(4)^ | -0.152 | 0.121 | -0.391 | 0.073 | 1.571 | 1 | 0.210 | -0.199 | -0.066 |
| DAS^(5)^ | -0.268 | 0.121 | -0.496 | -0.046 | 4.685 | 1 | 0.030 | -0.332 | -0.214 |
| SRP.rank_avg^(6)^ | -0.112 | 0.075 | -0.257 | 0.021 | 2.180 | 1 | 0.140 | -0.157 | -0.060 |

^(1)^ not indicated because of having a very limited interpretation, respectively

^(2)^ z-transformed to a mean of zero and a standard deviation (sd) of one; mean and sd of the original variable were 5.536 and 1.190, respectively

^(3)^ log- and then z-transformed; mean and sd of the original log-transformed variable were 1.469 and 0.569, respectively

^(4)^ log- and then z-transformed; mean and sd of the original log-transformed variable (+1) were 0.219 and 0.225, respectively

^(5)^ log- and then z-transformed; mean and sd of the original log-transformed variable (+1) were 0.488 and 0.292, respectively

^(6)^ z-transformed to a mean of zero and a standard deviation (sd) of one; mean and sd of the original variable were 0.536 and 0.345, respectively

## Further analysis of the effect of individual subjects

The models described above do not account for non-independence of the observations due to the individuals present, partly overlapping in different unique group compositions. However, it seems plausible to assume that the presence or absence of certain individuals might influence the outcome of a trial. We hence fitted an additional model complementing for the analysis considering the number of successful trials per session (model 2a). The model was essentially identical to the one described above with the exception that it included an additional random intercepts effect for the identities of the individuals present in the trial and random slopes of all predictors within them. The model was fitted with a function written by Roger Mundry. This function estimates the fixed and random effects (individual specific Best Linear Unbiased Predictors (BLUPS; (Baayen, 2008)) and their standard deviations using maximum likelihood and was tested with simulated data comprising 'normal' random effects (comprising only one level per observation) and comparing its results with that of the function glmmTMB (package glmmTMB version 1.0.0; Brooks et al. 2017). These tests showed results very similar (but not identical) to those of glmmTMB when using a negative binomial error distribution (but not when using a binomial error distribution) but only when the contributions of the random effects were not too low. The crucial difference between glmmTMB and function we used here is that summarizes BLUPs per observation before determining the log-likelihood. Since the function was not tested extensively, the results it reveals should be treated cautiously.

The additional model including additional random effects for the individuals present revealed essentially identical results with the exception that it also revealed a negative effect of maximum rank distance on the number of successful trials (estimate +/- SE=-0.372+/-0.144, z=-2.588, P=0.010).

SI table 6 Number of trials in which subjects obtained x amount of rewards by individual; average is the mean number of rewards subject obtained over all trials.

| Rewards | Jo | Fr | Pa | Ke | Ly | Wy | Pu | An |
| --- | --- | --- | --- | --- | --- | --- | --- | --- |
| 0 | 5 | 2 | 7 | 9 | 23 | 14 | 14 | 36 |
| 1 | 9 | 8 | 31 | 33 | 103 | 44 | 30 | 74 |
| 2 | 187 | 321 | 378 | 370 | 219 | 239 | 272 | 328 |
| 3 | 121 | 67 | 49 | 56 | 12 | 23 | 9 | 59 |
| 4 | 37 | 7 | 3 | 8 | 1 | 5 | 1 | 10 |
| 5 | 3 | 0 | 0 | 1 | 0 | 0 | 0 | 0 |
| Average | 2.51 | 2.17 | 2.02 | 2.05 | 1.62 | 1.88 | 1.86 | 1.87 |

SI table 7 Number of trials in which subjects obtained x amount of rewards by rank in group; average is the mean number of rewards subject of specific rank in group obtained over all trials.

| Rewards | 1st | 2nd | 3rd | 4th |
| --- | --- | --- | --- | --- |
| 0 | 8 | 11 | 30 | 61 |
| 1 | 12 | 71 | 123 | 126 |
| 2 | 538 | 641 | 592 | 543 |
| 3 | 199 | 77 | 54 | 66 |
| 4 | 47 | 7 | 7 | 11 |
| 5 | 3 | 0 | 1 | 0 |
| Average | 2.34 | 2.00 | 1.86 | 1.80 |
